# Temporal Trends in Dietary Sodium Intake Among Adults Aged ≥19 Years — United States, 2003–2016

**DOI:** 10.15585/mmwr.mm7042a4

**Published:** 2021-10-22

**Authors:** Lasha S. Clarke, Katherine Overwyk, Marlana Bates, Soyoun Park, Cathleen Gillespie, Mary E. Cogswell

**Affiliations:** ^1^Division for Heart Disease and Stroke Prevention, National Center for Chronic Disease Prevention and Health Promotion, CDC; ^2^IHRC, Inc., Atlanta, Georgia; ^3^Panum Group LLC, Bethesda, Maryland.

Hypertension, which can be brought on by excess sodium intake, affects nearly one half of U.S. adults and is a major risk factor for heart disease, the leading cause of death in the United States (*1*). In 2019, the National Academies of Sciences, Engineering, and Medicine (NASEM) established the Chronic Disease Risk Reduction (CDRR) intake, a chronic-disease–specific recommendation for dietary sodium of 2,300 mg/day. Reducing daily sodium to CDRR intake is expected to reduce chronic disease risk among healthy persons, primarily by lowering blood pressure (*2*). Although the 2019 sodium CDRR intake is equivalent in number to the 2005 Tolerable Upper Limit (UL) released by NASEM (then known as the Institute of Medicine), the UL was intended to provide guidance on safe intake levels, not to serve as an intake goal (*2*). To describe excess sodium intake in the context of the CDRR intake goal, this report analyzed National Health and Nutrition Examination Survey (NHANES) data from 2003 to 2016 to yield temporal trends in usual sodium intake >2,300 mg/day and in mean sodium intake, unadjusted and adjusted for total energy intake, among U.S. adults aged ≥19 years. The percentage of U.S. adults with sodium intake above CDRR intake was 87.0% during 2003–2004 and 86.7% during 2015–2016. Among U.S. adults overall, no significant linear trend was noted from 2003 to 2016 in unadjusted or energy intake–adjusted mean sodium intake. Small, significant declines were observed in mean usual sodium intake among some groups (adults aged 19–50 years, non-Hispanic White adults, adults experiencing obesity, and adults without hypertension). However, after energy adjustment, only adults aged ≥71 years and Mexican American adults demonstrated significant change in usual sodium intake. Many U.S. adults might be at risk for chronic disease associated with sodium intake above CDRR intake, and efforts to lower sodium intake could improve population cardiovascular health. The results of this report support enhanced efforts to reduce population sodium intake and cardiovascular disease risk, including the Food and Drug Administration’s (FDA’s) recently released guidance for the reduction of sodium in the commercially processed, packaged, and prepared food supply.*

NHANES, a series of cross-sectional surveys of nationally representative samples of the noninstitutionalized U.S. civilian population, uses a documented multistage design methodology^†^ to release data in 2-year cycles. This investigation used data collected from U.S. adults aged ≥19 years with valid dietary data, as determined by National Center for Health Statistics criteria,^§^ during seven NHANES cycles from 2003–2004 to 2015–2016. Among 70,059 persons examined during 2003–2016, 40,544 were aged ≥19 years. Among those, 4,312 (11%) participants with incomplete or missing dietary recall data were excluded, yielding an analytic sample of 36,232. Unweighted response rates among adults aged ≥20 years examined during that period range from 53.8% (2015–2016) to 70.9% (2009–2010).^¶^

Findings were stratified by sex, age group (19–30, 31–50, 51–70, and ≥71 years), race and ethnicity (non-Hispanic White, non-Hispanic Black, and Mexican American), obesity status (has or does not have obesity, defined by body mass index),** and hypertension status (has or does not have hypertension, defined by 2017 hypertension guidelines).^††^ Race- and ethnicity-specific results are reported for Mexican American persons as opposed to all Hispanic persons because of consistent oversampling among Mexican American persons over time.^§§^

Details on sodium intake assessment using NHANES have been published elsewhere (*3*–*5*). To estimate mean usual sodium intake, unadjusted and adjusted for energy intake and excess sodium intake (i.e., >2,300 mg/day) (*2*), this report used the National Cancer Institute method^¶¶^ for usual intake estimation along with post-stratified adjusted balanced repeated replication weights. Up to two complete and reliable 24-hour dietary recalls*** were used for estimation of the percentage of persons above or below the intake threshold. Energy intake–adjusted sodium intake (i.e., sodium intake adjusted for total calories consumed) was estimated using the residual method (*6*,*7*) and was calculated as the sum of the expected intake for a participant with mean energy intake and the residual from the linear regression model.

To assess the statistical significance of temporal trends in sodium consumption above CDRR intake and in mean usual sodium intake, this report treated survey cycle as a continuous variable in linear regression models. The inverse value of the estimated variance from post-stratified weights was used to account for the complex survey design. All statistical analyses were performed using SAS-callable SUDAAN (version 11.0.3; RTI International), and p-values <0.05 were considered statistically significant. This activity was reviewed by CDC and was conducted consistent with applicable federal law and CDC policy.^†††^

During 2003–2016, the percentage of U.S. adults aged ≥19 years whose sodium intake exceeded 2,300 mg/day ranged from 86.7% (2015–2016) to 89.2% (2011–2012) (Table 1). No significant linear trends were found among the overall population or any subgroups. Mean usual sodium intake, unadjusted for energy intake, was 3,521 mg/day during 2003–2004 and 3,468 mg/day during 2015–2016, the 2-year cycle with the lowest intake (Table 2). Temporal declines in mean usual sodium intake (average decline per 2-year cycle) were statistically significant among adults aged 19–30 years (30 mg), 31–50 years (45 mg), non-Hispanic White adults (33 mg), adults experiencing obesity (36 mg), and adults not experiencing hypertension (29 mg). Mean usual energy intake–adjusted sodium intake among U.S. adults aged ≥19 years was lowest (3,333 mg/day) during 2003–2004, and was 3,464 mg/day during 2015–2016 (Table 3). This intake decreased significantly by an average of 33 mg during every 2-year survey cycle among adults aged ≥71 years (p<0.01) and increased significantly by an average of 60 mg per survey cycle among Mexican American adults (p<0.01). 

**TABLE 1:** Percentage of adults with usual sodium intake >2,300 mg/day, by survey years and selected characteristics — National Health and Nutrition Estimation Survey, United States, 2003–2016

Characteristic	Survey yrs, % (SE)							Percentage point change every 2 yrs	p-value for linear trend*
	2003–2004	2005–2006	2007–2008	2009–2010	2011–2012	2013–2014	2015–2016		
**Overall**	**87.0 (0.6)**	**88.6 (0.4)**	**87.8 (0.6)**	**89.0 (0.5)**	**89.2 (0.6)**	**87.7 (0.5)**	**86.7 (0.5)**	**−0.13**	**0.52**
**Sex**									
Women	77.4 (1.1)	80.6 (0.7)	80.2 (1.0)	82.8 (0.9)	82.6 (0.7)	78.9 (0.6)	77.8 (0.8)	−0.22	0.63
Men	96.6 (0.3)	97.6 (0.3)	96.7 (0.3)	97.1 (0.3)	97.3 (0.4)	96.8 (0.4)	95.8 (0.3)	−0.14	0.23
**Age group, yrs**									
19–30	94.1 (1.1)	90.2 (1.4)	91.0 (1.4)	91.9 (0.7)	94.5 (1.1)	93.4 (1.0)	87.7 (0.9)	−0.50	0.34
31–50	89.5 (1.1)	91.5 (0.5)	89.4 (0.9)	91.0 (0.8)	92.2 (0.8)	89.4 (0.8)	90.7 (0.9)	−0.08	0.74
51–70	84.9 (1.3)	88.5 (0.8)	88.4 (1.1)	89.5 (0.8)	85.9 (1.1)	85.0 (1.0)	86.2 (0.6)	−0.42	0.25
≥71	68.5 (1.2)	78.3 (1.5)	77.3 (1.3)	75.5 (0.9)	77.1 (1.6)	76.4 (1.6)	74.2 (1.8)	0.92	0.26
**Race/Ethnicity^†^**									
NH, Black	83.2 (1.3)	83.9 (1.8)	85.5 (1.2)	85.5 (1.5)	87.3 (0.7)	81.4 (1.2)	83.6 (1.3)	0.05	0.93
NH, White	88.3 (0.8)	90.9 (0.4)	88.9 (0.6)	90.6 (0.6)	89.1 (0.8)	88.4 (0.4)	86.7 (0.8)	−0.43	0.13
Mexican American	83.8 (1.7)	80.6 (1.3)	84.0 (1.2)	81.6 (1.1)	96.0 (0.8)	90.6 (1.4)	88.1 (1.3)	1.85	0.16
**Has obesity^§^**									
Yes	90.0 (0.9)	89.6 (0.5)	88.7 (1.2)	89.5 (0.5)	90.4 (1.1)	86.5 (0.8)	87.1 (0.9)	−0.51	0.06
No	85.9 (0.7)	88.6 (0.6)	88.1 (0.8)	89.0 (0.7)	88.8 (0.6)	88.8 (0.4)	86.4 (0.7)	0.14	0.57
**Has hypertension^¶^**									
Yes	85.1 (0.9)	88.4 (0.4)	86.5 (0.7)	88.4 (0.6)	87.2 (0.7)	84.4 (0.6)	87.3 (0.9)	−0.34	0.37
No	89.7 (0.6)	89.3 (0.6)	89.4 (0.9)	89.6 (0.7)	90.9 (0.7)	90.4 (0.6)	86.3 (0.6)	−0.25	0.42

**Abbreviations**: BMI = body mass index; NH = non-Hispanic; SE = standard error.

* Based on the F-value; post-stratified balanced repeated replication weights were used to account for the complex survey design.

^†^ Persons of other or multiple races not reported; percentages will not sum to 100.

^§^ Obesity status is categorized based on clinical guidelines (https://www.cdc.gov/obesity/adult/defining.html) for categorizing BMI (kg/m^2^), such that “Not having obesity” corresponds with a BMI <30, and “Having obesity” corresponds with a BMI ≥30.

^¶^ Hypertension status is based on mean blood pressure and self-reported use of antihypertensive medications and is defined using the 2017 Hypertension Guidelines (https://www.acc.org/~/media/Non-Clinical/Files-PDFs-Excel-MS-Word-etc/Guidelines/2017/Guidelines_Made_Simple_2017_HBP.pdf), where “Having hypertension” is defined as mean systolic blood pressure ≥130 mmHg, mean diastolic blood pressure ≥80 mmHg, or self-reported use of antihypertensive medication. For the purpose of this analysis, participants who did not meet the 2017 Hypertension Guidelines definition for hypertension were defined as “Not having hypertension.”

**TABLE 2:** Mean usual sodium intake among adults, by survey years and selected characteristics — National Health and Nutrition Estimation Survey, United States, 2003–2016

Characteristic	Survey yrs, mg/day (SE)							Avg. change in sodium (mg) every 2 yrs	p-value for linear trend*
	2003–2004	2005–2006	2007–2008	2009–2010	2011–2012	2013–2014	2015–2016		
**Overall**	**3,521 (17)**	**3,650 (20)**	**3,567 (28)**	**3,567 (20)**	**3,523 (12)**	**3,477 (16)**	**3,468 (22)**	**−17**	**0.13**
**Sex**									
Women	2,992 (20)	3,057 (26)	3,014 (19)	2,978 (16)	2,961 (14)	2,966 (13)	2,974 (18)	−9	0.10
Men	4,093 (28)	4,311 (31)	4,186 (43)	4,205 (32)	4,131 (22)	4,035 (25)	3,996 (37)	−31	0.15
**Age group, yrs**									
19–30	3,888 (52)	3,850 (43)	3,769 (47)	3,769 (48)	3,760 (42)	3,740 (34)	3,666 (53)	−30	<0.01
31–50	3,731 (34)	3,898 (19)	3,775 (36)	3,729 (27)	3,741 (28)	3,636 (21)	3,617 (27)	−45	0.01
51–70	3,257 (34)	3,464 (24)	3,419 (38)	3,474 (32)	3,346 (37)	3,310 (29)	3,409 (30)	−4	0.81
≥71	2,722 (23)	2,924 (25)	2,866 (35)	2,872 (31)	2,904 (22)	2,973 (30)	2,917 (35)	31	0.06
**Race/Ethnicity^†^**									
NH, Black	3,268 (37)	3,430 (48)	3,373 (31)	3,312 (32)	3,415 (26)	3,420 (38)	3,240 (36)	0.3	0.99
NH, White	3,581 (21)	3,715 (21)	3,623 (31)	3,622 (22)	3,520 (18)	3,468 (18)	3,466 (29)	−33	0.05**^§^**
Mexican American	3,474 (37)	3,348 (48)	3,351 (30)	3,316 (62)	3,762 (49)	3,613 (76)	3,633 (28)	44	0.08
**Has obesity^¶^**									
Yes	3,579 (42)	3,741 (35)	3,598 (39)	3,589 (29)	3,533 (31)	3,438 (33)	3,507 (36)	−36	0.04
No	3,507 (26)	3,629 (19)	3,566 (28)	3,561 (25)	3,536 (15)	3,511 (16)	3,447 (27)	−18	0.10
Has hypertension**									
Yes	3,379 (29)	3,617 (35)	3,507 (33)	3,451 (22)	3,472 (22)	3,393 (22)	3,448 (29)	−8	0.59
No	3,656 (26)	3,692 (25)	3,596 (26)	3,664 (25)	3,574 (24)	3,548 (18)	3,490 (25)	−29	0.01

**Abbreviations**: BMI = body mass index; NH = non-Hispanic; SE = standard error.

* Based on the F-value; post-stratified balanced repeated replication weights were used to account for the complex survey design.

^†^ Persons of other or multiple races not reported; percentages will not sum to 100.

^§^ P-value = 0.048.

^¶^ Obesity status is categorized based on clinical guidelines (https://www.cdc.gov/obesity/adult/defining.html) for categorizing BMI (kg/m^2^), such that “Not having obesity” corresponds to a BMI <30, and “Having obesity” corresponds to a BMI ≥30.

** Hypertension status is based on mean blood pressure and self-reported use of antihypertensive medications and is defined using the 2017 Hypertension Guidelines (https://www.acc.org/~/media/Non-Clinical/Files-PDFs-Excel-MS-Word-etc/Guidelines/2017/Guidelines_Made_Simple_2017_HBP.pdf), where “Having hypertension” is defined as mean systolic blood pressure ≥130 mmHg, mean diastolic blood pressure ≥80 mmHg, or self-reported use of antihypertensive medication. For the purpose of this analysis, participants who did not meet the 2017 Hypertension Guidelines definition for hypertension were defined as “Not having hypertension.”

**TABLE 3:** Mean usual energy intake–adjusted sodium intake among adults, by survey years and selected characteristics — National Health and Nutrition Estimation Survey, United States, 2003–2016

Characteristic	Survey years, mg/day (SE)							Avg. change in sodium (mg) every 2 yrs	p-value for linear trend*
	2003–2004	2005–2006	2007–2008	2009–2010	2011–2012	2013–2014	2015–2016		
**Overall**	**3,333 (13)**	**3,524 (14)**	**3,524 (9)**	**3,480 (10)**	**3,432 (6)**	**3,427 (13)**	**3,464 (18)**	**−3**	**0.86**
**Sex**									
Women	3,299 (19)	3,477 (16)	3,444 (13)	3,381 (12)	3,339 (13)	3,341 (12)	3,395 (13)	−9	0.51
Men	3,371 (15)	3,582 (24)	3,616 (18)	3,588 (15)	3,531 (12)	3,527 (20)	3,535 (31)	21	0.31
**Age group, yrs**									
19–30	3,308 (31)	3,470 (23)	3,540 (23)	3,477 (21)	3,451 (26)	3,476 (19)	3,525 (39)	13	0.36
31–50	3,327 (24)	3,585 (16)	3,538 (17)	3,493 (14)	3,478 (16)	3,472 (22)	3,504 (17)	−2	0.89
51–70	3,394 (19)	3,536 (23)	3,526 (21)	3,505 (20)	3,401 (11)	3,380 (17)	3,441 (27)	−15	0.34
≥71	3,258 (22)	3,425 (21)	3,455 (19)	3,386 (22)	3,336 (26)	3,353 (21)	3,317 (26)	−33	<0.01
**Race/Ethnicity^†^**									
NH, Black	3,366 (13)	3,556 (16)	3,540 (13)	3,492 (11)	3,398 (8)	3,421 (13)	3,431 (23)	−7	0.70
NH, White	3,202 (31)	3,397 (20)	3,423 (12)	3,310 (22)	3,361 (12)	3,344 (16)	3,387 (24)	−4	0.77
Mexican American	3,154 (39)	3,192 (32)	3,282 (16)	3,288 (25)	3,468 (29)	3,407 (38)	3,516 (29)	60	<0.01
**Has obesity^§^**									
Yes	3,410 (22)	3,668 (23)	3,593 (25)	3,556 (10)	3,483 (14)	3,462 (20)	3,526 (26)	−11	0.59
No	3,300 (17)	3,460 (14)	3,494 (11)	3,439 (16)	3,404 (9)	3,411 (15)	3,424 (20)	−1	0.96
**Has hypertension^¶^**									
Yes	3,340 (13)	3,560 (17)	3,556 (15)	3,463 (10)	3,449 (13)	3,431 (18)	3,457 (20)	6	0.74
No	3,318 (18)	3,500 (19)	3,486 (10)	3,494 (14)	3,409 (12)	3,431 (19)	3,469 (20)	2	0.88

**Abbreviations**: BMI = body mass index; NH = non-Hispanic; SE = standard error.

* Based on the F-value; post-stratified balanced repeated replication weights were used to account for the complex survey design.

^†^ Persons of other or multiple races not reported; percentages will not sum to 100.

^§^ Obesity status is categorized based on clinical guidelines (https://www.cdc.gov/obesity/adult/defining.html) for categorizing BMI (kg/m^2^), such that “Not having obesity” corresponds with a BMI <30, and “Having obesity” corresponds with a BMI ≥30.

^¶^ Hypertension status is based on mean blood pressure and self-reported use of antihypertensive medications and is defined using the 2017 Hypertension Guidelines (https://www.acc.org/~/media/Non-Clinical/Files-PDFs-Excel-MS-Word-etc/Guidelines/2017/Guidelines_Made_Simple_2017_HBP.pdf), where “Having hypertension” is defined as mean systolic blood pressure ≥130 mmHg, mean diastolic blood pressure ≥80 mmHg, or self-reported use of antihypertensive medication. For the purpose of this analysis, participants who did not meet the 2017 Hypertension Guidelines definition for hypertension were defined as “Not having hypertension.”

## Discussion

Most U.S. adults consume dietary sodium above CDRR intake and could benefit from sodium reduction to lower their chronic disease risk. Among U.S. adults overall and in most subgroups evaluated, sodium intake remained similar from 2003 to 2016, with approximately 95% of men and 77% of women at increased cardiovascular disease risk because of excess intake during 2015-2016. Although total sodium intake gives a measure of the health risk, adjusting for energy intake allows better understanding of whether temporal changes in intake are associated with the amount of food consumed versus the amount of sodium in those foods. Slight but significant linear temporal declines in mean usual sodium intake among some subgroups were not significant after adjusting for energy intake, suggesting that decreases in the calories consumed over time might explain the trend, as opposed to decreases in the amount of sodium within foods. After energy intake adjustment, which avoids the confounding effect of energy intake (*8*), the amount of sodium consumed over time declined among adults aged ≥71 years and increased among Mexican American adults.

Data from What We Eat in America^§§§^ support shifts in energy intake. Total mean energy intake among adults decreased from 2,216 kcal/day during 2003–2004 to 2,105 kcal/day during 2015–2016. During the same period, average daily energy intake increased among men (by 146 kcal) and women aged ≥70 years (by 50 kcal). During 2003–2004, mean energy intake among Mexican American persons was 2,386 kcal/day. During 2015–2016, the data are presented for all Hispanic persons (because of sampling methodology implemented in NHANES 2009–2010)^¶¶¶^; Hispanic persons consumed 2,179 kcal/day on average. Additional reports suggest significant nonlinear declines in energy intake from 2003–2004 to 2009–2010 among U.S. adults aged 20–74 years, and among several subgroups (*9*). Thus, temporal changes in sodium intake observed in this study could be attributable to some changes in the amounts of sodium added by manufacturers to some foods, or the types and amounts of sodium-dense foods consumed from 2003–2004 to 2015–2016, which warrants further investigation. Recent FDA guidance, supported by evidence that most dietary sodium comes from processed and packaged and prepared foods, provides voluntary sodium reduction targets for manufactured foods, and foods prepared by commercial establishments, such as restaurants, to achieve in the next 2.5 years.

The findings from this study update previous reports on temporal trends in sodium intake and provide additional information relative to CDRR intake and temporal trends in sodium consumption accounting for energy intake (*3*,*4*,*6**,**10*). Differences in subgroup categorization and analytic methods preclude direct comparison; however, the results here are consistent with previous reports which used the sodium UL to illustrate that most U.S. adults consume >2,300 mg of sodium per day (*2*–*4*,*7*).

Given that higher sodium intake is positively correlated with higher total energy intake,**** assessment of energy intake–adjusted sodium intake is recommended to account for differences in energy intake across groups and over time (*6*,*8*). This report used the residual method to adjust for energy intake, consistent with a previous report (*6*). In addition, a large, nationally representative sample of U.S. adults with two complete dietary recalls was analyzed with measurement error methods to account for within-person day-to-day variability in all analyses. This analysis also considered changes in NHANES methodology for estimating sodium intake (i.e., the discontinuation of adjusting for optional salt added to eligible foods), allowing for more accurate description of trends from 2003 to 2016 (*3*–*5*). Previous research supports that excluding the salt adjustment step when using 24-hour dietary recall yields sodium intake estimates that are similar to estimates based on urinary sodium (*5*).

The findings in this report are subject to at least two limitations. First, the use of self-reported dietary information is subject to random error and social desirability biases. However, use of two 24-hour recalls and inclusion of energy intake adjustment account for some types of measurement error, including that attributable to day-to-day variability in sodium consumption (*6*). Further, given the prevalence of excess sodium consumption among U.S. adults, it is unlikely that time trends in the underreporting of sodium intake have meaningfully changed the conclusions drawn. Second, because the 2017 guideline (which was not released when data were collected during 2003–2016) was used to define hypertension and includes persons with undiagnosed hypertension, some persons categorized as having hypertension might have been unaware of their status.

Overall, this report provides updated information on temporal trends in sodium intake among U.S. adults. These data can update and support national strategies, including Healthy People 2030 objectives,^††††^ and recent FDA guidance to decrease sodium intake, lower hypertension risk, and improve population cardiovascular health.

**Summary**

**What is already known about this topic?**

U.S. adults’ usual sodium intake consistently exceeds national guidelines. Given associations between excess sodium intake and hypertension risk, the Chronic Disease Risk Reduction (CDRR) intake of 2,300 mg/day was recently established. Sodium consumption below this level is expected to reduce chronic disease risk.

**What is added by this report?**

During 2003–2016, ≥86% of U.S. adults consumed sodium above CDRR intake. Significant changes in unadjusted mean usual sodium intake were seen among some groups; other groups demonstrated significant changes only after energy intake adjustment.

**What are the implications for public health practice?**

Many U.S. adults might be at risk for chronic disease associated with sodium intake above CDRR intake. Efforts to lower sodium intake could improve population cardiovascular health.

## Footnotes

*
www.fda.gov/sodiumreduction

† https://wwwn.cdc.gov/nchs/nhanes/continuousnhanes/manuals.aspx?BeginYear=2019

§
https://www.cdc.gov/nchs/nhanes/measuring_guides_dri/measuringguides.htm

¶
https://wwwn.cdc.gov/nchs/nhanes/ResponseRates.aspx#response-rates

**
https://www.cdc.gov/obesity/adult/defining.html

††
https://www.acc.org/~/media/Non-Clinical/Files-PDFs-Excel-MS-Word-etc/Guidelines/2017/Guidelines_Made_Simple_2017_HBP.pdf

§§
https://wwwn.cdc.gov/nchs/nhanes/analyticguidelines.aspx

¶¶
https://epi.grants.cancer.gov/diet/usualintakes/method.html

***
https://dietassessmentprimer.cancer.gov/approach/table.html

††† 45 C.F.R. part 46.102(l)(2), 21 C.F.R. part 56; 42 U.S.C. Sect. 241(d); 5 U.S.C. Sect. 552a; 44 U.S.C. Sect. 3501 et seq.

§§§
https://www.ars.usda.gov/northeast-area/beltsville-md-bhnrc/beltsville-human-nutrition-research-center/food-surveys-research-group/docs/wweia-data-tables/

¶¶¶
https://www.ars.usda.gov/ARSUserFiles/80400530/pdf/0910/Table_2_NIN_RAC_09.pdf

****
https://www.who.int/elena/titles/guidance_summaries/sodium_intake/en/

††††
https://health.gov/healthypeople/objectives-and-data/browse-objectives/nutrition-and-healthy-eating/reduce-consumption-sodium-people-aged-2-years-and-over-nws-12
